# Editorial: Homeostasis and physiological regulation in the aquatic animal during osmotic stress

**DOI:** 10.3389/fphys.2022.977185

**Published:** 2022-08-08

**Authors:** Xingkun Jin, Xiaodan Wang, William Ka Fai Tse, Yan Shi

**Affiliations:** ^1^ Department of Marine Biology, College of Oceanography, Hohai University, Nanjing, China; ^2^ Laboratory of Aquaculture Nutrition and Environmental Health, School of Life Sciences, East China Normal University, Shanghai, China; ^3^ Laboratory of Developmental Disorders and Toxicology, Center for Promotion of International Education and Research, Faculty of Agriculture, Kyushu University, Fukuoka, Japan

**Keywords:** climate change, evolution, homeostasis, osmoregulation, salinity stress

An aquatic animal (either vertebrate or invertebrate) comes into intimate biological interactions with surrounding water media for most or all of its lifetime. Salinity is a pivotal environmental factor influencing the survival, growth and reproduction of aquatic animal. Often, the intensity, duration and frequency of this modulation work synergistically with temperature. This raises concerns about how and to what extent the osmotic stress that anthropogenic global warming will induce and bring impact on the aquatic animals. To cope with such stress, the aquatic animals had evolved various osmoregulatory and/or osmoconforming strategies that actively balance the absorption and secretion of water and/or ions to maintain osmotic homeostasis. Therefore, an effective osmoregulatory capacity favours euryhaline aquatic animal through habitat expansion and adaptive radiation in a climate change scenario. Systematic research focusing on how such intricate mechanisms evolve and function will not only contribute to the in-depth understanding of the physiology of osmoregulation, it also is of great significance for the practical optimization of selective breeding programs and environmental management in aquaculture of fish and aquatic invertebrates, and for the conservation of global fisheries.

In the current Research Topic of Aquatic Physiology, a section of the journals **Frontiers in Physiology** and **Frontiers in Marine Science**, a sum of six contributing articles have been included, encompassing physiological studies of osmotic-stressed fish and aquatic invertebrates that are of great economic importance in aquaculture and fishery. These studies provide integrative knowledge within and across levels of organization of life including molecule, tissue and organism, and have further implications in understanding osmoregulation in aquatic animals.

## Physiological responses to osmotic stress in select teleost fish

Appropriate environmental temperature and salinity both are critical to maintain the physiological homeostasis of aquatic organisms, therefore, thermal stress and salinity fluctuation would have consequential impact on the feeding behavior, digestion, metabolism, and ultimately the growth performance of aquatic animals. Chang et al. investigated physiological responses of gill ionocytes in freshwater-and seawater-acclimated milkfish, *Chanos chanos*, under hypothermal stress. Their study illustrated two different osmoregulatory strategies of energy metabolism in freshwater-and seawater-acclimated milkfish under hypothermal stress, and provided new insights into the osmoregulatory plasticity and its associations with cold endurance in euryhaline teleost species.

In addition to the direct interactions with the ambient environment as the major exterior organ, gill also plays crucial roles acclimating fish to the prevailing environmental changes with neuroendocrine epithelial cells. Aruna et al. investigated physiological responses of brain and gills in black porgy, *Acanthopagrus schlegelii,* transferred from seawater to freshwater. Through assessing the spatial-temporal transcriptional dynamics of stress-responsive peptide hormone, corticotropin releasing hormone (crh), its receptor (crhr), and sodium-potassium adenosine triphosphatase (a-nka) post *in-vivo* hypotonic stress and CRH-peptides injection, respectively, the authors demonstrated the bioregulatory functions of crh system (crh/crhr) in brain and gill in response to osmotic stress.

Physiological adaptability and regulatory versatility to environmental changes in salinity are of vital importance for teleost fishes, particularly for anadromous species that commonly encounter surrounding osmotic stress to a varying degree. Wang et al. showed physiological and transcriptomic responses of gill, kidney and intestine respectively in another euryhaline teleost species, *Takifugu obscurus*, upon hypertonic stress. Through integrative analyses of histological features and RNA-Seq profiles, the authors showed the tissue-specific micrographic alterations in the cellular and subcellular structure and activated molecular signaling pathways between freshwater-and brackish water-acclimated pufferfish. Their study provided histological and molecular evidences of osmoregulation under hypertonic stress in anadromous teleost.

## Physiological responses to osmotic stress in select invertebrates

Unlike the vertebrates, most marine invertebrates including bivalves and crustaceans are osmoconformers, of which the extracellular osmolality in body fluid (e.g., hemolymph) can be coordinatively modulated with the external osmolality, thereby minimizing the osmotic pressure difference between the organisms and the environment in an energy efficient manner. Sun et al. reported on the physiological responses upon hypotonic and hypertonic stresses in two breeds of marine oysters species, *Pinctada fucata*, with red and black shell color, respectively. The authors demonstrated slightly yet significantly different ionic concentrations in hemolymph and several metabolism-related enzymatic activities in gill and liver between oyster breeds varied in shell colors, respectively, under the hypotonic and particularly hypertonic stress. Wherein, the red-shelled oyster breed can respond promptly and acclimate efficiently to the environmental changes in salinity, indicating a superior osmoregulatory capacity in coping with osmotic stress thereof.

Among other organic osmolytes, marine invertebrates utilize many different free amino acids as one of the major osmotic effectors to promptly adjust tissue osmotic gradient, in the process of which the associated organic osmolytes transporters can be modulated accordantly. Lin et al. assessed physiological responses in the gill and mantle of a marine clam, *Meretrix lusoria*, under hypertonic stress. The authors reported significant accumulation and maintenance of ions and, to a lesser extent of free amino acids, in the tested tissues under hyperosmotic conditions. Furthermore, markedly elevated yet shortly restored transcription of ion and osmolyte transporters, respectively, were also observed. Altogether, their study provided physiological and molecular evidences of osmoregulation and osmoconformation in marine bivalves.

In freshwater or brackish-water habitats, aquatic animals including invertebrates are obliged to be able to cope with hydration issues caused by the dilute surrounding environment, that poses different selective pressures on the osmoregulation, metabolisms and growth of aquatic animals. Xue et al. investigated transcriptomic responses in the gill and hepatopancreas respectively of a brackish-and fresh-water shrimp, *Macrobrachium nipponense*, under a long-term hypertonic stresses. Through integrative analyses of RNA-Seq profiles and histological features of biomarkers, the authors showed that transcription of osmoregulatory and metabolic genes respectively associated to several signaling pathways, particularly, ion transport, oxidative phosphorylation, and glycometabolism were dramatically augmented to an increasing degree as the environmental salinity levels rise, and provide further insights into the trade-off between osmoregulation and energy metabolisms in aquatic invertebrates.

